# Association between Age at Type 2 Diabetes Onset and Diabetic Retinopathy: A Double-Center Retrospective Study

**DOI:** 10.1155/2023/5919468

**Published:** 2023-01-23

**Authors:** Mengdie Chen, Yiyun Wang, Ping Feng, Yao Liang, Qiao Liu, Mengyao Yang, Chaoyin Lu, Penghua Shi, Jian Cheng, Anjing Ji, Qidong Zheng

**Affiliations:** ^1^Department of Endocrinology, Taizhou Central Hospital (Taizhou University Hospital), Taizhou 318000, China; ^2^Department of Internal Medicine, The Second People's Hospital of Yuhuan, Yuhuan 317600, China

## Abstract

**Background:**

With the decreasing age of type 2 diabetes mellitus (T2DM) onset, the incidence of diabetic complications is gradually increasing. We evaluated the independent effect of age at diabetes onset on diabetic retinopathy (DR) development.

**Methods:**

A total of 7472 patients with T2DM were enrolled in the National Metabolic Management Center from September 2017 to May 2022. Anthropometry data, laboratory reports, and medical history were collected. The independent association of DR with age at diabetes onset was analyzed using multivariable logistic regression models. In addition, a stratified analysis was performed to determine the effect of confounding variables.

**Results:**

Of the 7472 patients recruited, 1642 (21.98%) had DR. Patients with DR had considerably younger ages of diabetes onset than those without DR (45 (38–53) years vs. 50 (43–57) years, *P* < 0.001). The proportion of patients with T2DM onset at a younger age was higher in the DR group than that in the non-DR group. Participants were divided into four groups according to their age at diabetes onset, namely, ≥60, <40, 40–49, and 50–59 years. Compared with patients with diabetes onset at age ≥ 60 years, those with diabetes onset at <40 years (odds ratio (OR): 5.56, 95% confidence interval (CI): 3.731–8.285, *P* < 0.001), 40–49 years (OR: 2.751, 95% CI: 2.047–3.695, *P* < 0.001), and 50–59 years (OR: 1.606, 95% CI: 1.263–2.042, *P* < 0.001) were at an increased risk of DR after adjusting for potential confounding factors. Furthermore, stratification analyses demonstrated that young age at diabetes onset is an independent risk factor for DR.

**Conclusions:**

Compared with diabetes onset at an older age, diabetes onset at a younger age is associated with a significantly increased DR risk.

## 1. Introduction

Diabetes mellitus is a serious and growing problem in terms of its effect on public health. According to a recent survey, 451 million individuals globally had diabetes in 2017, and 693 million will have diabetes by 2045 [1]. Historically regarded as a disease of middle- and older-age individuals, type 2 diabetes mellitus (T2DM) is increasingly being detected in younger patients [2]. T2DM onset at a younger age is more aggressive and more likely to cause severe complications than T2DM onset at an older age.

Diabetic retinopathy (DR) is a prevalent chronic complication of diabetes that is usually regarded as a major source of vision loss globally [3–5]. The prevalence rate of DR among the Chinese population is 1.14% [3], whereas the incidence rate of DR-related blindness is 7.7% [6]. DR can result in a decline in life quality and an increase in the economic burden on individuals and nations. It is also crucial to underline that cardiovascular risk, the major cause of death in diabetic patients, is increased in those with microvascular complications of diabetes. DR is one of the microvascular complications of diabetes. Therefore, DR could reduce life expectancy via raising the risk of cardiovascular diseases in diabetes [7]. Consequently, prevention, early detection, and appropriate management of DR are crucial for enhancing public health and reducing healthcare costs [8].

In recent decades, accumulative research has been undertaken to better comprehend the pathogenic characteristics of DR. Several factors have been elucidated about DR development, such as hyperglycemia, a long duration of diabetes, old age, female sex, hypertension, high lipid levels, anemia, and microalbuminuria [5, 9, 10].

Growing evidence suggests that younger age at diabetes onset may be an underlying risk factor for DR. Several studies have investigated the association between the age at diabetes onset and DR [11–16]. Some studies have revealed an association between young age at diabetes onset and an increased risk of DR [11–13], whereas others have found a decreased risk of DR or no influence of diabetes onset age on DR [14–16]. The effect of age at diabetes onset on DR remains unexplored. Therefore, we investigated 7472 patients with T2DM to determine the association between the age at diabetes onset and DR risk.

## 2. Materials and Methods

### 2.1. Participants

**Figure 1:**
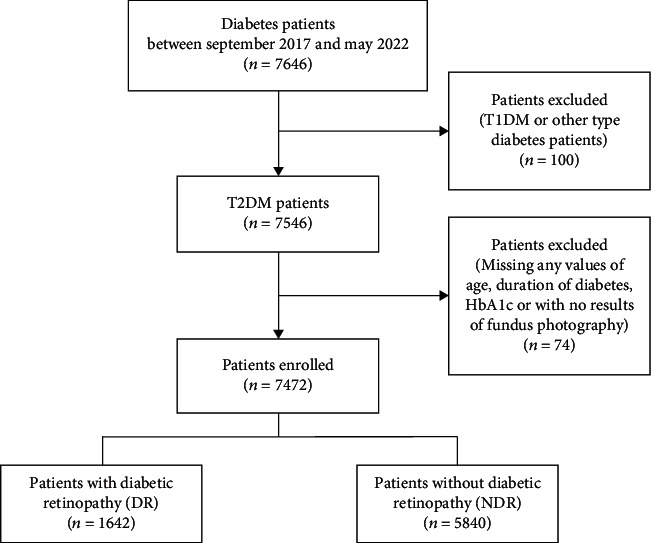
Flowchart of the study. Abbreviations: T1DM: type 1 diabetes mellitus; T2DM: type 2 diabetes mellitus; HbA1c: glycated hemoglobin.

From September 2017 to May 2022, we screened 7646 patients with diabetes from the National Metabolic Management Center (MMC) [17] in The Second People's Hospital of Yuhuan and Taizhou Central Hospital (Taizhou University Hospital). Patients with type 1 diabetes mellitus or other types of diabetes (*n* = 100) and those with missing data for age, diabetes duration, glycated hemoglobin (HbA1c), or fundus photography (*n* = 74) were excluded from the study. In total, 7472 individuals were enrolled in the present study. Then, 1642 T2DM patients with DR and 5840 without DR were selected as cases (DR) and controls (NDR), respectively. The study's flowchart is depicted in Figure 1. The ethics committees at the recruitment hospitals approved the study protocol. All participants gave their written informed consent.

### 2.2. Data Collection

A comprehensive clinical evaluation was performed on each individual. All data were collected using a dedicated MMC electronic medical record system [17]. The following data were collected: demographic variables (sex, age, and education level), diagnosis (diabetes and DR), medical history (diabetes duration, family history of diabetes, hypertension, and dyslipidemia), lifestyle factors (smoking and drinking), physical examination results (height, weight, and blood pressure), and laboratory test results, including fasting blood glucose (FBG), fasting serum C peptide, HbA1c, total cholesterol (TC), triglycerides (TG), high-density lipoprotein cholesterol (HDL-C), low-density lipoprotein cholesterol (LDL-C), serum creatinine, and the urinary albumin-to-creatinine ratio (UACR). The tests were conducted in the respective hospitals from which the patients were recruited. All the participants were evaluated for DR by using a DL-based system [18] that categorized the fundus images as a DR stage or ungradable due to image quality concerns. Ophthalmologists assessed the hardcopy of the fundus photographs collected from all participants. After at least 5 min of rest, blood pressure was recorded using electronic blood pressure monitors with the patient in a sitting posture. The participants were asked to wear lightweight clothes and remove their shoes before measuring their height and body weight. Body mass index (BMI) was calculated as weight (kg)/height (m)^2^. Homeostasis model assessment of insulin resistance (HOMA-IR) was calculated as 1.5 + (fasting C peptide (pmol/L) × fasting glucose (mmol/L))/2800 [19]. The estimated glomerular filtration rate (eGFR) was calculated using the equation of the Chronic Kidney Disease Epidemiology Collaboration [20].

### 2.3. Classification and Definition

Diabetes diagnosis was based on the World Health Organization guidelines of 1999 [21]. The participants were divided into four groups according to the age at diabetes onset as follows: <40, 40–49, 50–59, and ≥60 years. DR diagnosis was made by ophthalmologists based on the characteristic alterations of the retina caused by diabetes as observed in the fundus photographs. Hypertension was defined as systolic blood pressure (SBP) ≥ 140 mmHg, diastolic blood pressure ≥ 90 mmHg, or self-reported previous diagnosis of hypertension by a physician and taking antihypertensive drugs during the preceding 2 weeks [22]. Dyslipidemia was defined as one or more of the following conditions: TC ≥ 5.7 mmol/L, TG ≥ 1.7 mmol/L, LDL-C ≥ 3.6 mmol/L, and HDL-C < 1.29 mmol/L in women and <1.03 mmol/L in men [23]. The drinking status was classified as “yes” if participants consumed alcohol weekly or nearly weekly. The participants' smoking status was categorized as “yes” if they smoked daily or nearly daily. Education level was classified as below high school or high school and above.

### 2.4. Statistical Analysis

Continuous and categorical variables are presented as median (interquartile range, IQR) and frequency (%), respectively. Demographic and clinical features were compared using a chi-squared test and Mann–Whitney *U* test for categorical and continuous data, respectively. Multivariable logistic regression was used to evaluate the association between the age at diabetes onset and DR risk, with the >60 years group as the reference. Three models were used to adjust potential confounding factors: model 1: adjusted for age and sex; model 2: adjusted for variables in model 1 plus BMI, HbA1c, diabetes duration, and HOMA-IR; and model 3: adjusted for all variables in model 2 plus UACR, eGFR, education level, family history of diabetes, lifestyle factors (both smoking and drinking status), history of hypertension, history of dyslipidemia, and hypoglycemic drug use. We further performed a stratified analysis on the association between the age at diabetes onset and DR with subgroups of sex, age, HbA1c, and diabetes duration by using multivariable logistic regression models with full adjustment in model 3, unless stratified. All data were analyzed using SPSS 23.0 (IBM). A two-sided *P* value of <0.05 was considered statistically significant.

## 3. Results

### 3.1. Demographic and Clinical Characteristics of Participants

**Figure 2:**
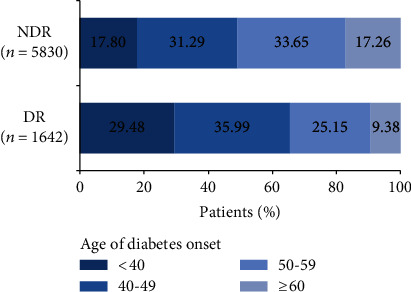
Distribution of the age of diabetes onset in patients with diabetic retinopathy (DR) and without diabetic retinopathy (NDR).

The demographic and clinical characteristics of the 7472 participants are presented in Table 1. The median (IQR) age of the participants was 55 (48–63) years, and 4465 (59.76%) of them were men. The median (IQR) age at diabetes onset was 49 (41–56) years. In total, 1642 (21.98%) patients received a diagnosis of DR. Patients with DR had considerably younger median (IQR) ages of diabetes onset than those without DR (45 (38–53) years vs. 50 (43–57) years, *P* < 0.001). The proportions of patients with diabetes onset at the age of <40, 40–49, 50–59, and ≥60 years were 29.48%, 35.99%, 25.15%, and 9.38% in the DR group, whereas they were 17.80%, 31.29%, 33.65%, and 17.26% in the NDR group, respectively (*P* < 0.001, Figure 2). Compared with the participants in the NDR group, those in the DR had a long duration of diabetes; high FBG, HbA1c, SBP, and UACR; and low fasting serum C peptide, HOMA-IR, BMI, and eGFR (all *P* < 0.001, Table 1). The participants in the DR group more frequently had a family history of diabetes and less frequently used hypoglycemic agents (all *P* < 0.001, Table 1) than those in the NDR group. Differences in age, sex, education level, smoking and drinking status, history of hypertension, and history of dyslipidemia across the groups were not statistically significant (Table 1).

### 3.2. Association between the Age at Diabetes Onset and DR Risk

Three models were used to investigate the relationship between the age at diabetes onset and DR risk (Table 2). Compared with patients with T2DM onset at the age of ≥60 years, those with T2DM at the age of <40 (odds ratios (ORs): 16.044, 95% confidence interval (CI): 12.049–21.362), 40–49 (OR: 5.654, 95% CI: 4.501–7.103), and 50–59 (OR: 2.372, 95% CI: 1.919–2.932) years were at a significantly high risk of DR after adjustment for sex and age (model 1). A similar pattern of adjusted ORs was seen when more variables (models 2 and 3) were accounted for (Table 2). The increased risk of DR was attenuated (OR: 6.573, 3.166, and 1.73 for onset ages < 40, 40–49, and 50–59 years, respectively) after further adjustment for BMI, HbA1c, diabetes duration, and HOMA-IR (model 2). The risk of DR slightly decreased after additional adjustments for UACR, eGFR, education level, family history of diabetes, smoking and drinking status, history of hypertension, history of dyslipidemia, and hypoglycemic drug use, but the overall association remained substantial (model 3, OR of DR: 5.56, 2.751, and 1.606 for onset ages < 40, 40–49, and 50–59 years, respectively).

### 3.3. Stratified Analysis for the Effects of the Age at Diabetes Onset on DR Risk

**Figure 3:**
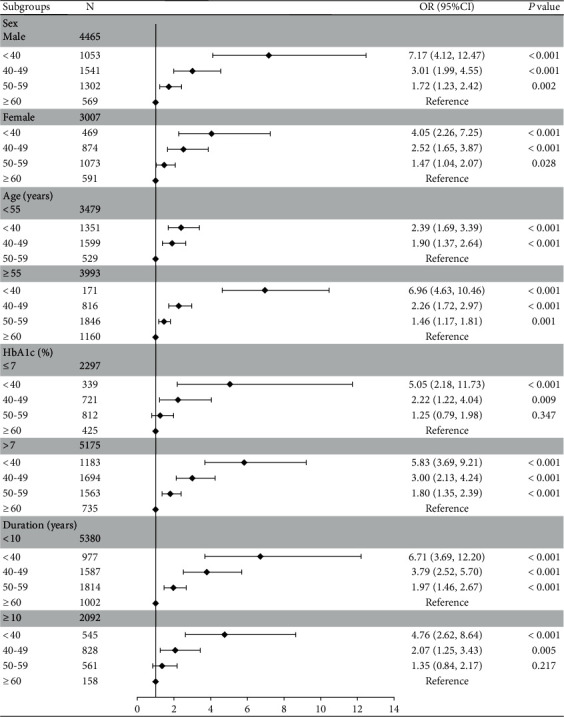
Subgroup analyses of the association of the age of diabetes onset with diabetic retinopathy. Adjusted for age, sex, BMI, HbA1c, diabetes duration, HOMA-IR, UACR, eGFR, education level, family history of diabetes, lifestyle factors (both smoking and drinking status), history of hypertension, history of dyslipidemia, and the use of hypoglycemic drugs, if not stratified. Abbreviations: OR: odds ratio; CI: confidence interval; BMI: body mass index; HbA1c: glycated hemoglobin; HOMA-IR: homeostasis model assessment for insulin resistance; UACR: urine albumin-to-creatinine ratio; eGFR: estimated glomerular filtration rate.

The relationship between the age at diabetes onset and the risk of DR was further investigated with stratified analysis in four subgroups, namely, sex (male or female), age (<55 or ≥55 years), HbA1c (≤7% or >7%), and diabetes duration (<10 or ≥10 years). The model was completely adjusted for variables in model 3 unless stratified. The stratified analysis revealed a favorable connection between the age at diabetes onset and the risk of DR in all subgroups (Figure 3), which was consistent with the result overall. In each model, younger age at diabetes onset was associated with a greater risk of DR. However, no significant effect of diabetes onset was observed in the age group of 50–59 years on the risk of DR in the patients with HbA1c ≤ 7% (*P* = 0.347) and diabetes duration ≥ 10 years (*P* = 0.217; Figure 3).

## 4. Discussion

In this retrospective study, we demonstrated that the age at diabetes onset was significantly earlier in patients with DR than in those without DR. We discovered an increase in DR prevalence among individuals with diabetes onset at a younger age. The effect of the age at diabetes onset on DR risk is the greatest in patients diagnosed with diabetes at <40 years of age. This result remained significant after adjustments for other traditional DR risk variables, demonstrating that diabetes onset at a young age is an independent risk factor for DR development. When we further stratified the patients based on sex, age, HbA1c, and diabetes duration, we discovered that the increased prevalence of DR in patients with diabetes onset at a young age persists. Of particular interest, subgroup analysis indicated that participants in the group of <40 years with male or older age (age ≥ 55 years) had significantly enhanced DR risk compared with the group of ≥60 years. Therefore, we should pay more attention to those patients in the follow-up process. However, no statistical difference was observed between the group of 50–59 years on the risk of DR in the patients with HbA1c ≤ 7% as well as diabetes duration ≥ 10 years and the group of ≥60 years. This suggests that good glycemic control and a longer duration of T2DM may mitigate the risk effect of onset age on the incidence of DR.

Some studies have indicated that the prevalence of DR was higher in early-onset diabetes than in late-onset diabetes [24–26]. A prospective study revealed that young age at T2DM onset (15–40 years) increased the incidence twice as compared with T2DM onset at a later age (60–70 years) after adjustments for multiple risk factors [13]. A retrospective review of 3568 patients with T2DM through a propensity score-matched (PSM) cohort analysis was conducted in two different diabetes-onset age groups (ages 40 and 60 years), which suggested that patients categorized in both the early-onset disease groups had a higher risk of developing DR before and after PSM [27]. Yuan et al. [11] found that diabetes onset at an early age increased the risk of DR in patients with T2DM duration of 10–20 years and HbA1C ≥ 7%. An Asian multinational study with 41029 patients with diabetes demonstrated that DR risk was higher among those diagnosed with diabetes earlier (<40 years) than in those diagnosed later (≥50 years) [12]. These results support the conclusions drawn from our research, which is that diabetes onset at a younger age was associated with DR risk. However, a Chinese study involving 29442 patients with T2DM found that a significant risk of DR in the early-onset group was attributed to a longer duration of diabetes [14]. A cross-sectional study found that the age at diabetes onset did not significantly associate with an increased risk of DR after adjustments for confounding variables [15]. The results of this study are similar to those of the UKPDS [28] and ADVANCE [29] trials. According to the UKPDS trial, older age at diabetes diagnosis was associated with DR. The ADVANCE trial reported that microvascular events were associated with only diabetes duration and not age or diabetes-onset age. Furthermore, a longitudinal observational cohort study found that the age at diabetes onset could not predict the risk of DR at 17 years of diabetic duration [16]. In addition, Hillier and Pedula [30] reported a decreased risk of DR in those who received a diagnosis of diabetes at an earlier age. The discordant results may be due to variability in population characteristics, differences in stratification based on the age at diabetes onset, and discrepancies in adjusting for the main risk variables among different studies. As revealed by our sensitivity analysis, no significant effect of diabetes-onset age on the risk of DR was observed in the 50–59 years age-group patients with HbA1c ≤ 7% and duration ≥ 10 years.

The potential mechanisms by which diabetes onset at a young age increases DR risk are unclear. Compared with patients with late-onset T2DM, those with early-onset T2DM tend to have a more aggressive metabolic profile with the early development of *β*-cell dysfunction and insulin resistance [9] and more prolonged exposure to hyperglycemic conditions. These patients have experienced metabolic syndrome for a longer while before developing diabetes. They have suffered from obesity for a long time, with raised leptin levels, inflammation, and insulin resistance with reduced adipokine levels, and they may have developed nonalcoholic fatty liver disease which has a bidirectional relationship with diabetes. In addition, it is also possible that due to their younger age, they are less likely to perform scheduled visits to detect diseases [31]. The increased risk of DR associated with young age at T2DM onset can be explained by the aforementioned factors [32]. Despite adjusting the diabetes duration and HbA1c levels, our study indicated that diabetes onset at a young age is an independent risk factor for DR. This shows that patients with diabetes onset at a young age are predisposed to DR by extra variables. Studies have already offered insights that the levels of vascular endothelial growth factor (VEGF) [33] and growth hormone [34, 35] play a crucial role in DR development and have been reported to vary with age at diabetes onset [33]. Moreover, early adulthood is characterized by a more robust response to VEGF and the highest levels of circulating growth hormone [13, 36]. The sensitivity of ocular VEGF to hyperglycemia can be stronger in younger people. Therefore, diabetes mellitus in youth could be predisposed to DR development and an increased rate of DR. Some studies recently pointed out that in patients with both albuminuria and chronic kidney disease (CKD), the albumin excretion rate (AER) and eGFR are strongly associated with DR [37, 38]. It is why our results showed that the risk of DR slightly decreased after additional adjustments for UACR and eGFR. Furthermore, other multiple factors such as early life determinants, diet, obesity, physical activity, socioeconomic factors, and family history may be involved in the pathogenesis [2]. Therefore, further research is necessary to uncover the true underlying mechanisms.

This study has several limitations. First, this was a retrospective study, and therefore, the cause of DR could not be directly assessed. We used statistical adjustments to decrease the effect of diabetes duration and other risk factors, but we could not rule out all potential confounding variables for DR, such as the medication history. It is clear now that newer antidiabetic drugs present several pleiotropic effects which involve endothelial dysfunction amelioration. Given that DR represents one of the microvascular complications of diabetes, the amelioration of the endothelial function may play a role in reducing DR incidence [39]. Second, patients included in this study were hospital-based, and the study involved only a Chinese population recruited from two institutions, which may have resulted in a selection bias. Third, due to the insidious start of this disease, it is difficult to pinpoint the exact age of T2DM onset. The diabetes diagnosis may be at an older age than the onset age. In the future, multicenter population-based prospective research with large sample sizes should be necessary. For the present, mydriatic fundus oculi are still the gold standard to diagnose DR, and they should be involved in our future study.

## 5. Conclusions

In summary, our study demonstrates that a younger age at T2DM onset is associated with a considerably increased DR risk. Therefore, the age at T2DM onset may be of significance in the screening, prevention, and management of this chronic disease. To delay the formation and progression of DR in patients with T2DM onset at a young age, intensive glycemic management and frequent DR monitoring are required early on.

## Figures and Tables

**Table 1:** Demographic and clinical parameters of study patients.

Variables	Total (*n* = 7472)	DR (*n* = 1642)	NDR (*n* = 5830)	*P* value
Age (y)	55 (48-63)	56 (49-63)	55 (48-63)	0.709
Male, *n* (%)	4465 (59.76)	963 (58.65)	3502 (60.07)	0.305
Duration of diabetes (y)	4.17 (0.83-10.5)	9.33 (3.42-13.69)	3.17 (0.33-9.42)	<0.001
Onset age of diabetes (y)	49 (41-56)	45 (38-53)	50 (43-57)	<0.001
SBP (mmHg)	130 (121-142)	132 (122-145)	130 (120-141)	<0.001
DBP (mmHg)	75 (69-83)	75 (69-83)	75 (69-82.75)	0.415
BMI (kg/m^2^)	25.17 (23.1-27.48)	24.78 (22.56-26.97)	25.30 (23.24-27.64)	<0.001
Fasting blood glucose (mmol/L)	8.14 (6.63-10.89)	8.98 (6.9-12.17)	7.97 (6.58-10.55)	<0.001
Fasting serum C peptide (ng/mL)	2.1 (1.5-2.8)	1.82 (1.22-2.55)	2.16 (1.57-2.9)	<0.001
HOMA-IR	3.62 (2.89-4.60)	3.53 (2.71-4.55)	3.64 (2.93-4.63)	<0.001
HbA1c (%)	8 (6.8-9.8)	8.7 (7.3-10.6)	7.8 (6.7-9.6)	<0.001
TG (mmol/L)	1.46 (1.01-2.24)	1.43 (0.99-2.15)	1.47 (1.02-2.25)	0.056
TC (mmol/L)	5.08 (4.29-5.92)	5.03 (4.26-5.88)	5.09 (4.30-5.93)	0.134
LDL-C (mmol/L)	1.13 (0.95-1.35)	1.11 (0.92-1.35)	1.13 (0.95-1.35)	0.001
HDL-C (mmol/L)	2.88 (2.24-3.56)	2.85 (2.21-3.54)	2.89 (2.25-3.57)	0.412
eGFR	103.64 (84.42-125.12)	100.78 (79.86-125.27)	104.25 (85.77-124.97)	<0.001
UACR (mg/g)	16.83 (7.36-49.54)	28.05 (10.23-98.04)	15.22 (6.87-40.27)	<0.001
High school education and above, *n* (%)	1230 (16.46)	253 (15.41)	977 (16.76)	0.2
Family history of diabetes, *n* (%)	3638 (48.69)	864 (52.62)	2774 (47.58)	<0.001
History of hypertension, *n* (%)	3582 (47.94)	794 (48.36)	2788 (47.82)	0.716
History of dyslipidemia, *n* (%)	6151 (82.32)	1350 (82.22)	4801 (82.35)	0.913
Smoking, *n* (%)	1824 (24.41)	377 (22.96)	1447 (24.82)	0.126
Drinking, *n* (%)	1032 (13.81)	206 (12.55)	826 (14.17)	0.097
Hypoglycemic drugs, *n* (%)	4573 (61.20)	1210 (73.69)	3363 (57.68)	<0.001

Note: data are expressed as median (interquartile range) or *n* (%). The groups were compared using the chi-squared test and Mann–Whitney *U* test for categorical and continuous data, respectively. Abbreviations: NDR: nondiabetic retinopathy; DR: diabetic retinopathy; SBP: systolic blood pressure; DBP: diastolic blood pressure; BMI: body mass index; HOMA-IR: homeostasis model assessment for insulin resistance; HbA1c: glycated hemoglobin; TG: triglycerides; TC: total cholesterol; HDL-C: high-density lipoprotein cholesterol; LDL-C: low-density lipoprotein cholesterol; eGFR: estimated glomerular filtration rate; UACR: urine albumin-to-creatinine ratio.

**Table 2:** Association of the age of diabetes onset with diabetic retinopathy in patients with type 2 diabetes based on multivariate logistic regression analysis.

	Model 1	*P* value	Model 2	*P* value	Model 3	*P* value
	OR (95% CI)		OR (95% CI)		OR (95% CI)	
<40	16.044 (12.049-21.362)	<0.001	6.573 (4.472-9.66)	<0.001	5.56 (3.731-8.285)	<0.001
40-49	5.654 (4.501-7.103)	<0.001	3.166 (2.382-4.208)	<0.001	2.751 (2.047-3.695)	<0.001
50-59	2.372 (1.919-2.932)	<0.001	1.73 (1.371-2.183)	<0.001	1.606 (1.263-2.042)	<0.001
≥60	Reference		Reference		Reference	

Note: the analysis was performed using multivariable logistic regression. Model 1: adjusted for age and sex; model 2: adjusted for variables in model 1 plus BMI, HbA1c, diabetes duration, and HOMA-IR; model 3: adjusted for variables in model 2 plus UACR, eGFR, education level, family history of diabetes, lifestyle factors (both smoking and drinking status), history of hypertension, history of dyslipidemia, and hypoglycemic drug use. Abbreviations: OR: odds ratio; CI: confidence interval; BMI: body mass index; HbA1c: glycated hemoglobin; HOMA-IR: homeostasis model assessment for insulin resistance; UACR: urine albumin-to-creatinine ratio; eGFR: estimated glomerular filtration rate.

## Data Availability

The data used to support the findings of this study are available from the corresponding author upon reasonable request.
